# Role of Pectoralis Muscle Analysis in Breast Magnetic Resonance Imaging for Body Composition Evaluation Before and After Neoadjuvant Chemotherapy for Breast Cancer

**DOI:** 10.3390/nu17101698

**Published:** 2025-05-16

**Authors:** Annarita Pecchi, Francesca Mogavero, Sara Zanni, Davide Vaccari, Riccardo Cuoghi Costantini, Fabio Canino, Federico Piacentini, Roberto D’Amico, Massimo Dominici, Pietro Torricelli

**Affiliations:** 1 Division of Radiology, Department of Medical and Surgical Sciences of Children and Adults, University of Modena and Reggio Emilia, 41224 Modena, Italy; annarita.pecchi@unimore.it (A.P.); 307994@studenti.unimore.it (F.M.); pietro.torricelli@unimore.it (P.T.); 2 Integrated Diagnostic Imaging Department of Modena, Azienda USL of Modena, 41121 Modena, Italydavidevaccari262@gmail.com (D.V.); 3 Division of Clinical Statistics, Department of Medical and Surgical Sciences of Children and Adults, University of Modena and Reggio Emilia, 41224 Modena, Italy; riccardo.cuoghicostantini@unimore.it (R.C.C.); roberto.damico@unimore.it (R.D.); 4 Division of Oncology Department of Medical and Surgical Sciences of Children and Adults, University of Modena and Reggio Emilia, 41224 Modena, Italy; fabio.canino@unimore.it (F.C.); federico.piacentini@unimore.it (F.P.); massimo.dominici@unimore.it (M.D.)

**Keywords:** breast cancer, sarcopenia, pectoralis muscle, body composition, MRI, CT

## Abstract

**Background**: sarcopenia is a physical condition characterized by the loss of muscle mass and strength; it is associated with worse outcomes in oncological diseases and is recognized as an independent predictor of poor survival. The aim of our work is to evaluate the correlation between the pectoralis muscles area (PMA) calculated in breast MRI examinations and the body composition parameters assessed in CT examinations, in order to identify a threshold useful for diagnosing sarcopenia in breast cancer patients who are candidates for neoadjuvant chemotherapy (NACT), so as to be able to provide the correct nutritional counselling. **Methods**: we included 116 patients with non-metastatic breast cancer, who were studied with MRI before and after NACT, in the 2018–2023 period. All patients were categorized according to age, weight, height, and BMI. Using MRI scans, both before and after treatment, we measured the PMA at the level of the sternal angle of Louis and evaluated the changes caused by NACT, and we performed the same procedure for CT body composition parameters. **Results**: the ROC we calculated describes the ability of the PMA to discriminate sarcopenic patients from non-sarcopenic ones, identifying an optimal cut-off of 20.55, which achieves a specificity of 92%. The variations in PMA after NACT showed a strong, statistically significant association with the variations in all CT body composition parameters. **Conclusions**: these results introduce the possibility of also assessing body composition in breast MRI. The novelty in this study is to have estimated, on the basis of these correlations, a cut-off value that reflects the skeletal muscle index threshold for the definition of sarcopenia that is usually used.

## 1. Introduction

Sarcopenia has been defined as an age-related loss of skeletal muscle mass and function, and it is present in 6–22% of elderly adults. To diagnose sarcopenia, a finding of muscle quantity or quality loss is enough. Since several pathological conditions can lead to the onset of sarcopenia, even in patients under age 65, developing methods of determining muscle mass or function has become necessary [1,2,3]. The commonly accepted definition of sarcopenia is the one from the European Working Group (EWGSOP), which highlights the coexistence of reduced muscle mass and function. This definition was updated in 2018 (EWGSOP2), underlining the role of muscle strength loss as a prime indicator of probable sarcopenia [1,2,4]. The current literature shows how the assessment of muscle mass measurements can be a useful biomarker in clinical practice. For example, in patients awaiting surgery for cancer [5], sarcopenia has been linked to an increased risk of complications in the post-operative follow-up period, increased hospitalization and death, and chemotherapy- and radiotherapy-related adverse effects [6,7,8,9,10].

Computed tomography (CT) is considered the *gold standard* technique for body composition analysis thanks to its reproducibility, but it has the significant limitation of the radiation exposure, and for this reason, it is not useful as a sarcopenia screening tool in patients who do not need CT scans for other reasons [11,12,13]. A simple way to assess muscle mass is to calculate the psoas major muscle area on an axial plane passing through the level of the third or fourth lumbar vertebra, or the total skeletal muscle area (psoas muscles, paraspinal muscles, and abdominal wall muscles area at L3 or L4 level) [14,15,16,17].

Magnetic resonance imaging (MRI) is a good alternative to CT, although there are no approved protocols or measurement cut-off values. In postmortem studies, MRI provided data that could be easily compared to CT data, especially regarding muscle area, volume, and fatty infiltration [18,19,20]. The proton content difference between muscle and adipose tissue provides a high contrast, allowing accurate tissue measurements [17,21]. MRI provides further information regarding muscular tissue quality, being able to detect intramuscular edema or fibrous degeneration. Therefore, MRI can help to distinguish between normal and sarcopenic muscles, especially in elderly patients, and could be used especially in patients with breast cancer, using the pectoralis muscles to obtain an estimate of the total skeletal muscle mass [18,19,22]. A notable advantage of MRI over CT is its absence of radiation exposure; despite this, MRI has evident limitations, such as the absence of standardized protocols, extremely high costs, and image processing issues [23].

Breast cancer is the most common cancer in females, and it is one of the greatest causes of female mortality. In addition to the commonly recognized prognostic factors for breast cancer patients, body composition, and especially muscle and fat distribution, are increasingly important predictors of chemotherapy effectiveness and clinical outcomes [24,25]. Sarcopenia in patients with early breast cancer showed a strongly increased risk of death, up to 71%; sarcopenic patients with metastatic breast cancer had an increased risk of grade 3 and grade 4 toxicity related to chemotherapy [26]. In a recent meta-analysis of 5284 patients with early breast cancer, sarcopenia was associated with an increased risk of mortality (33%) and disease progression/recurrence (29%). In addition, sarcopenic patients were more likely to develop drug-related G3-4 toxicities during chemotherapy [26]. In addition, a recent prospective study showed that patients with early breast cancer who received neoadjuvant chemotherapy had a greater decline in postoperative nutritional status than those who received upfront surgery. This shows an increase in chemotherapy-induced catabolic processes, with an increased risk of perioperative complications and need for prolonged hospitalization [27]. Such data outlined the need to implement screening strategies. A particular condition, termed “sarcopenic obesity”, characterized by a combination of excess weight and reduced muscle mass, is strongly associated with impaired functional status and increased post-chemotherapy morbidity [22,28,29]. Obesity, especially when visceral fat is increased, is a known risk factor for cancer progression [22,30,31]. In breast cancer patients, CT is used in cases of positive axillary lymph nodes, higher T stages, histotypes with aggressive molecular features, and suspected metastasis [19,32].

As reported in other studies, such as in the work of Peter Teschann et al., sarcopenic obesity is associated in cancer patients with a worsening of outcomes such as overall survival and disease-free survival. In cancer patients, the simple evaluation of BMI is deficient, as reported by the same authors, while the analysis of body composition in CT or MR is more reliable as it is able to define with greater precision the distribution of the adipose and muscle compartments. The early identification of situations with greater risk in cancer patients, such as sarcopenic obesity, allows nutritional support to be through established correct nutrition and the optimal integration of physical activity, which are crucial factors [5,33,34,35].

Specifically, regarding breast cancer, Jodkiewicz M et al. have shown how nutritional counselling is essential from the time of tumour diagnosis before neoadjuvant treatment. Nutritional counselling provides important information on how to maintain or reduce body weight and achieve favourable changes in body composition, even in women with normal BMI, and reduce gastrointestinal disorders, which are side effects of cancer treatment. The authors also conclude by emphasizing how dietary counselling is a relatively low-cost tool that can significantly improve patient outcomes and quality of life [36]. Other authors have demonstrated how oral nutritional supplements can improve body composition and prevent hypoalbuminemia and lipid abnormalities in women with breast cancer undergoing chemotherapy [37].

Breast MRI is recognized by the main breast cancer guidelines and by numerous works in the literature as the method of choice for evaluating the response to neoadjuvant chemotherapy (NACT) in women with breast cancer [38,39]. Breast MRI can evaluate, with good accuracy, several crucial parameters such as tumour size, volume, and contrast enhancement, which are commonly used to define treatment response [19,28,32,40]. Recent studies demonstrated a significant correlation between MRI assessment of pectoralis major muscle area and psoas major muscle area; therefore, MRI could be used as a valuable tool for the assessment of muscle mass in breast cancer patients, even in the earliest stages of disease, when CT is not indicated [30]. MRI could, therefore, be used to monitor body composition changes potentially leading to adverse events and worse clinical outcomes [19,32,41,42], especially since chemotherapy is dosed according to body surface area calculated using BMI, and this could lead to under- or overestimation of metabolically active tissue.

The aim of our work is to evaluate the correlation between the pectoralis muscles area calculated on breast MRI examinations and the body composition parameters assessed on CT examinations in order to identify a MR threshold useful for diagnosing sarcopenia in breast cancer patients who are candidates for NACT, and also to create an instrument to precociously identify some at-risk conditions, which can be correct with the appropriate nutritional tools. We also evaluate the main changes in body composition parameters induced by chemotherapy.

## 2. Materials and Methods

This study was approved by the local ethics committee (Prot. AOU 0026512/23-08/09/2023). Informed consent was obtained from the included patients. In this observational retrospective trial, we included consecutive patients with non-metastatic breast cancer diagnosis, studied with breast MRI at the Modena Radiology Department before and after NACT, in the period 2018–2023.

Inclusion criteria:

- Breast cancer, histologically confirmed, candidate, from the multidisciplinary team to NACT.
- Availability of good-quality, artefact-free breast MRI before and after neoadjuvant treatment.
- Availability of histological data related to post-chemotherapy surgery.
- Chemotherapy protocol performed at our centre.

Exclusion criteria:

- Presence of breast implants.
- Proven claustrophobia.
- No breast MRI available both pre- and post-NACT.
- Poor image quality with artefacts.

All patients included were categorized according to age (<49, 50–69, >70 years), weight, height, and body mass index (BMI). For all patients, we collected histological data: histotype, prognostic immunohistochemistry, proliferative index, and baseline TNM staging.

We also considered the chemotherapy treatment regimen, and for each chemotherapy drug administered to each patient in pre-operative treatment, the following parameters were calculated:

- **Standard dose intensity (SDI)**: defined as the ratio of the total standard dose (mg/m^2^) to the total standard time required for administration (weeks).
- **Delivered dose intensity (DDI)**: defined as the ratio of the total dose actually received (mg/m^2^) to the total actual time required for administration (weeks).
- **Relative dose intensity (RDI)**: ratio of DDI to SDI × 100.

Finally, for each patient, the arithmetic mean of the RDIs for each individual chemotherapy drug received during the preoperative treatment was calculated, resulting in the **total RDI** (**tRDI**). A cut-off value of 90% was chosen to distinguish patients who received almost the full expected dose of chemotherapy per body surface area (≥90%) from those who received less than the expected dose (<90%).

### 2.1. Body Composition Analysis

#### 2.1.1. MR Imaging

All breast MRI scans were performed using 1.5 T scanners (Philips Ingenia, GE Healthcare Signa Voyager), with a coil dedicated for breast imaging. The breast MR study protocol included axial T1- and T2-weighted sequences; axial DWI, with four b-values (b/0, b/300, b/600, b/1000); axial 3D T1-weighted fat sat gradient echo, before and after IV injection of contrast medium.

**Figure 1:**
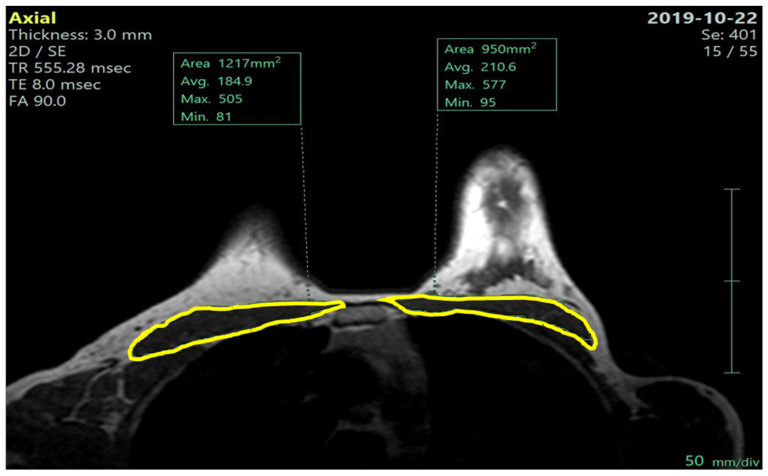
Pre-NACT left and right pectoralis muscle areas (MRI—axial T1 non-fat sat image).

**Figure 2:**
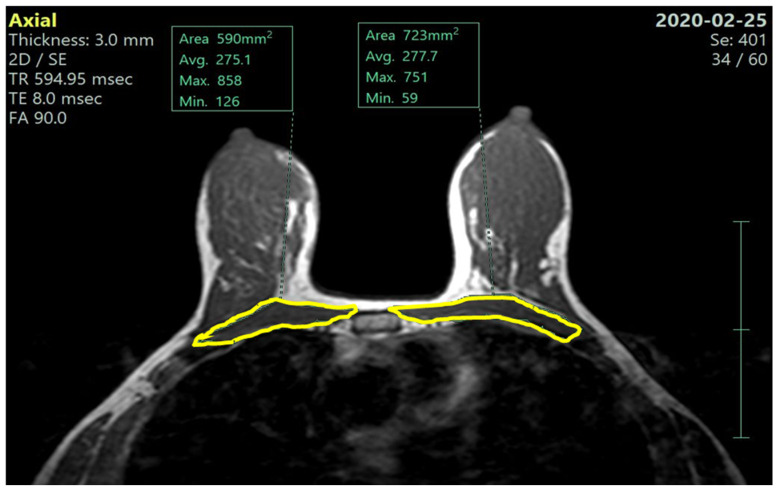
Post-NACT left and right pectoralis muscle areas (MRI—axial T1 non-fat sat image).

We measured, on both before- and after-NACT MRI scans, the whole pectoralis muscles area (PMA): we selected an axial image in the T1-weighted sequence at the level of the Louis angle (manubriosternal joint; second costal cartilage). On this reference image, using a dedicated software, we segmented the right and left pectoralis muscle along the muscle profile as shown in Figure 1 and Figure 2, automatically obtaining the pectoralis area, expressed in cm^2^.

We separately analyzed the left and right areas and the bilateral area, given by the sum of the two left and right areas.

Subsequently, we calculated the difference in the values of the pectoralis muscle areas (left, right, and bilateral) before and after chemotherapy (Δ_pect_).

The individual values of the pectoral muscle areas were normalized according to the height of the patients, expressed in square metres, thus obtaining the pectoralis muscle index (PMI), reported as cm^2^/m^2^.

#### 2.1.2. CT Imaging

We also considered the CT scans performed by patients upon diagnosis of breast cancer, when available. All CT scans were performed with GE Healthcare scanners (GE VCT Lightspeed, GE Optima), with a slice thickness of 2.5 mm and interval of 1.25 mm. We used the CT data to obtain the body composition parameters, which were processed using a dedicated software. We selected unenhanced cross-sectional images at the third lumbar vertebra (L3), where both transverse processes were clearly identifiable.

The software allows the selective visualization of muscle tissues, by establishing threshold values within a density range from −29 to +150 Hounsfield units (HU). This targeted visualization enabled a more accurate segmentation of skeletal muscle tissue. Regions of interest (ROI) were manually delineated using the software tool, aligning with the designated compartments for analysis. Within these ROIs, the software automatically computed the area expressed in square centimetres.

We considered:

- **Right, left and bilateral psoas muscle area** at the L3 level.
- **Psoas muscle index:** psoas muscle area normalized with respect to stature and reported as cm^2^/m^2^.
- **Skeletal muscle area (SMA)** (cm^2^): obtained manually tracing a ROI encompassing the psoas muscles, paraspinal muscles (erector spine, quadratus lumborum, multifidus), and wall muscles (transversus, internal and external oblique, rectus abdominis) at the L3 level.
- **Skeletal muscle index (SMI)**: cross-sectional SMA normalized with respect to stature and reported as cm^2^/m^2^.

The software allows also the selective visualization of adipose tissues, by establishing threshold values within a density range from −180 to −30 HU. This targeted visualization enabled a more accurate segmentation of adipose tissue. ROIs were manually delineated using the software tool, aligning with the designated compartments for analysis. Within these ROIs, the software automatically computed the area expressed in square centimetres. We considered:

- **Visceral fat area (VFA) (cm^2^)**: by manually tracing the visceral adipose tissue area after setting the adipose tissue HU range.
- **Visceral fat index (VFI) cm^2^/m^2^)**: visceral fat area normalized for height expressed in cm^2^/m^2^.
- **Subcutaneous fat area (SFA) (cm^2^)**: by manually tracing the subcutaneous adipose tissue area after setting the adipose tissue HU range.
- **Subcutaneous fat index (SFI) (cm^2^/m^2^)**: subcutaneous fat area was normalized for height and expressed in cm^2^/m^2^.
- **Total fat area (cm^2^):** the sum of the subcutaneous and visceral fat areas
- **Total fat index (cm^2^/m^2^):** the total fat area was normalized for height and expressed in cm^2^/m^2^.

For patients who had both pre- and post-treatment CT scans, we calculated the differences (Δ) in total skeletal muscle area, skeletal muscle index, and visceral fat index.

In line with previous studies [1,2], we also classified the patients into sarcopenic or not sarcopenic using a CT skeletal muscle index cut-off for sarcopenia in women of 39 cm^2^/m^2^. Using BMI values, we identified patients belonging to the subgroup with sarcopenic obesity.

### 2.2. Statistical Analysis

We performed a descriptive statistical analysis for each considered variable: for numerical variables, we reported mean, standard deviation, and range, while for categorical variables, we calculated absolute frequencies and percentages.

To investigate the relationships between patients’ characteristics, univariable generalized regression models were used. In particular, the associations between pectoralis muscle and body composition pre- and post-chemotherapy were investigated using linear regression models, and the obtained results were expressed as mean differences (MD). Linear regression models were also estimated to analyze the differences between body composition parameters with respect to the main chemotherapy regimens performed by the patients and the dose intensity parameters.

The associations between the pectoralis muscle parameters and sarcopenia were measured in terms of odds ratios (OR), estimated via logistic regression models. Finally, the association between the radiological parameters of body composition and the biological disease subtypes was analyzed using multinomial regression models, and the results were reported in terms of relative risk ratios (RRR).

All results were reported with 95% confidence intervals (CI) and *p*-values. For each linear model, the goodness of fit was assessed by inspecting residual plots. Moreover, the determination coefficient R^2^ was reported.

The ability of the pectoralis major area to approximate the classification into sarcopenia and non-sarcopenia obtained from SMI was investigated using the receiver operating characteristic curve (ROC) and the corresponding area under the curve (AUC). Youden’s statistic was used to identify an optimal value for the pectoralis major area to discriminate between sarcopenic and non-sarcopenic patients. The sensitivity and specificity of the optimal cut-off point were reported.

Results were considered statistically significant if the corresponding *p*-values were below the alpha level, set at 0.05.

Imputation of missing values was not performed; thus, each analysis was carried out on complete case data.

All statistical analyses were performed using R version 4.3.2 statistical software (the R Foundation for Statistical Computing).

## 3. Results

### 3.1. Population Characteristics

We included 116 female patients with non-metastatic breast cancer, whose general population characteristics are summarized in Table 1, below. The mean age was 55.80 and median age was 56, with a SD of 10.53; the minimum age was 36; and the maximum age was 79. BMI calculation resulted in a mean of 25.12 with a SD of 4.85 and a median of 24.71; the minimum value was 17.36, while the maximum was 42.32.

### 3.2. Histology

Histological analysis results are reported in Table 2:

### 3.3. Baseline Local Staging

Baseline local staging and baseline nodal staging are summarized in Table 3.

### 3.4. Chemotherapy

Regarding preoperative treatment, all the patients received treatment with taxane-based regimens, 84.5% of whom received paclitaxel. A total of 81.9% of patients received anthracyclines, while 21.5% received carboplatin as part of the treatment schedule, 52.6% received one or more monoclonal antibodies (trastuzumab ± pertuzumab, immuno-checkpoint inhibitors) in addition. About 20% of patients received four–six cycles of neoadjuvant treatment, compared to 80% who received up to eight cycles. On the other hand, 58 (52.7%) received ≤17 weeks of preoperative treatment, versus 52 (47.2%) who received >17 weeks. A total of 48 patients (41%) received neoadjuvant treatment with a dose-dense schedule (shorter intervals between one NACT treatment and another, at the same dosage). A total of 65 patients (59.1%) maintained a total relative dose intensity (tRDI) ≥ 90%. In the study population, pCR rates were 44% (51 out 116 patients). The drugs used in the neoadjuvant setting are summarized in Table 4:

### 3.5. Body Compositionanalysis

#### 3.5.1. MRI

Segmentation of the pectoralis major muscles area, performed on T1 MR images, provided the following values reported in Table 5:

#### 3.5.2. CT

A total of 85 out of 116 patients had a pre-treatment CT scan available; 31 patients also had a post-treatment CT scan available. Table 6 shows the body muscle and fat composition parameters of patients who were subjected to pre- and post-chemotherapy CT scans, obtained through third lumbar vertebra (L3) segmentation.

The variations in the pectoralis muscle detected between the two MRs were significant, as were the variations in the psoas muscle, total muscle area, and SMI normalized on height in the same patients.

### 3.6. Correlations

The correlation between the pectoralis areas (right, left, bilateral) pre- and post-chemotherapy and the pectoralis delta values showed a strong, statistically significant association with all body composition parameters, as shown in Table 7 and Table 8, both related to the muscular compartment and the adipose compartment both pre- and post-chemotherapy. The association is also confirmed to be significant among the parameters normalized with respect to the patients’ height.

In line with previous studies [1,2], we used a CT SMI cut-off for sarcopenia in women of 39 cm^2^/m^2^, according to which we classified the patients who underwent a pre-chemotherapy CT scan as follows:

- Sarcopenic: 73/85 patients (85.88%)
- Non-sarcopenic: 12/85 patients (14.12%)

We also considered the coexistence of sarcopenia with a BMI class of overweight or higher (BMI > 25), which was present in 32/85 patients (37.65%).

Using the same cut-off, we classified the patients who underwent a post-chemotherapy CT scan as follows:

- Sarcopenic: 30/31 patients (96.77%)
- Non-sarcopenic: 1/31 patients (3.23%)

Of these patients, 13/31 (41.93%) were in the overweight or higher BMI range (>25).

The pectoralis muscle parameters, pre- and post-NACT, pectoralis delta, and standardized pectoralis index were associated with the evidence of sarcopenia identified on the SMI in a statistically significant way (Table 9). They are also significantly associated with the condition of sarcopenic obesity detected in the post-treatment.

**Figure 3:**
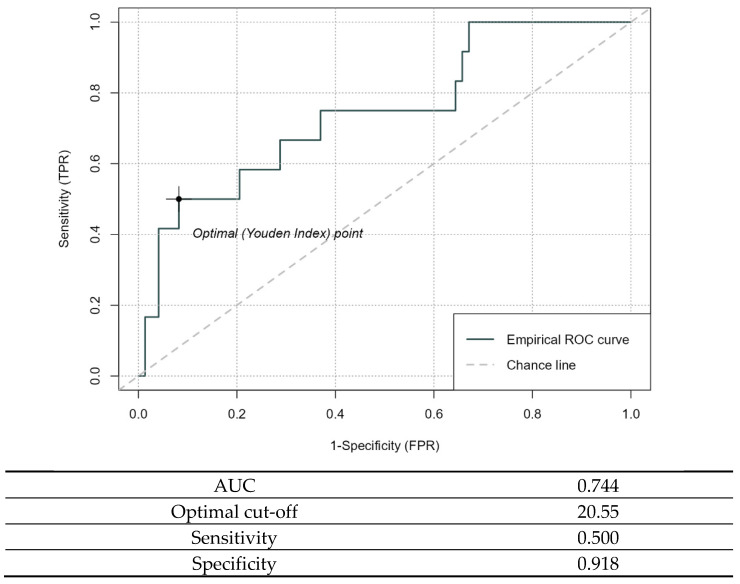
The ROC.

Among the 73 CT-proven sarcopenic patients, we measured, in MRI, a mean bilateral pectoralis muscle area of 14.88 cm^2^ (SD 3.98, minimum 8.25, maximum 30.91, median 14.36). Among the 12 remaining non-sarcopenic patients, we measured a mean bilateral pectoralis muscle area of 18.94 cm^2^ (SD 4.39, minimum 13.5, maximum 25.46, median 19.19). The ROC, shown below (Figure 3), describes the ability of the pectoralis major area to approximate the classification into sarcopenia and non-sarcopenia obtained from SMI, and has identified an optimal cut-off for the pectoralis major area of 20.55, which achieves a specificity of 92%. In Table 10 we classified the patients according to this cut off.

The association between the radiological parameters of body composition and the biological disease subtypes was analyzed: in patients with triple-negative disease, we recorded significantly lower pre-treatment values of right psoas muscle area (RRR = 0.62, 95% CI 0.41–0.95, *p* = 0.03), left (RRR = 0.67, 95% CI 0.46–0.97, *p* = 0.027) and bilateral (RRR = 0.79, 95% CI 0.64–0.97, *p* = 0.027) than in patients with luminal-like disease. A numerical, but not statistically significant, similar association exists when comparing HER2-positive and luminal-like patients: pre-treatment values of right psoas muscle area (RRR = 0.71, 95% CI 0.50–1.00, *p* = 0.053), left (RRR = 0.75, 95% CI 0.55–1.02, *p* = 0.069), and bilateral (RRR = 0.84, 95% CI 0.71–1.00, *p* = 0.052). A pre-surgical T2 tumour staging was associated with a greater reduction in bilateral pectoralis muscle area (Δ_pect_) (mean difference MD = −2.733, 95% CI −5.273–−0.193, *p* = 0.035). In our population, patients with HER2-positive disease were significantly associated with lower values of ∆ pectoralis muscle index (RRR = 0.48, 95% CI 0.24–0.97, *p* = 0.040) and ∆ pectoralis muscle bilateral area (RRR = 0.75, 95% CI 0.57–0.98, *p* = 0.035) than patients with luminal-like disease. Specifically, the mean of ∆ pectoralis muscle index and ∆ pectoralis muscle bilateral area were 0.65 (SD ± 0.51) and 1.72 (SD ± 1.33) in HER2-positive patients, 0.97 (SD ± 0.91) and 2.56 (SD ± 2.26) in luminal-like cases, with a statistically significant difference between the means of the two groups of −0.32 (95% CI −0.62 and −0.02, *p* = 0.039) and −0.83 (95% CI −1.60 and −0.07, *p* = 0.034), respectively. The duration, dose intensity, and density of the treatment schedule were also associated with these parameters. The use of a dose-dense schedule was significantly associated with more negative ∆ visceral fat area (OR = 0.94, 95% CI 0.88–0.90, *p* = 0.029) and ∆ visceral fat index (OR = 0.83, 95% CI 0.71–0.98, *p* = 0.027) values, resulting in increased visceral fat in patients receiving this regimen. Specifically, the mean of ∆ visceral fat area and ∆ visceral fat index were −12.71 (SD ± 16.07) and −4.76 (SD ± 6.01) in the dose-dense group, 5.09 (SD ± 20.61) and 2.05 (SD ± 7.68) in the non-dose-dense group, with a statistically significant difference between the means of the two groups of −17.8 (95% CI −31.25 and −4.34, *p* = 0.015) and −6.81 (95% CI −11.83 and −1.79, *p* = 0.013), respectively. The findings revealed no significant relation between the duration of preoperative therapy, as measured by both the number of cycles (four–six vs. eight cycles) and the length of treatment (≤17 vs. >17 weeks), and radiological body composition parameters. Similarly, no significant association was identified between the tRDI of chemotherapy received by patients (≥90% vs. <90%) and the aforementioned parameters.

## 4. Discussion

Sarcopenia is a physical condition characterized by the loss of muscle mass and strength [43]; there is a lot of evidence in the literature that associates sarcopenia with worse outcomes in oncological diseases, recognizing it as an independent predictor of poor survival, associating it with a higher incidence of chemotherapy toxicity and lower response to treatments [5,10,30,31,33,34,35,44,45,46].

There are several works in the literature that underline the role of body composition assessed with CT or MR compared to BMI in the correct evaluation of the adipose and muscular compartments in order to identify risk conditions such as sarcopenia and sarcopenic obesity that can influence the toxicity of treatments and the outcome of oncological diseases [10,33,36].

Furthermore, there is a lot of evidence in the literature that underlines the role of correct nutritional intake, diet control, and physical activity, established already at the beginning of the cancer patient’s treatment path, to improve body composition, reducing CT toxicity and improving oncological outcomes [34,35,36,37]. Nutritional counselling represents a simple, inexpensive tool that can positively intervene in different aspects of the cancer patient’s treatment path. From this perspective, body composition becomes an essential tool in defining and recognizing risk situations with greater precision than BMI and consistently during the oncological follow-up, in which to implement adequate nutritional strategies.

Breast MRI is recognized by the main breast guidelines, and by numerous works in the literature, as the method of choice for evaluating the response to NACT in women with breast cancer [38,39,47]. Some recent works have shown a correlation between the psoas muscle area measured on CT and the pectoralis muscle area measured on MR images [30]: these results introduce the possibility of considering the pectoral muscle area calculated in MRI as an indicator of the muscular status in patients with breast cancer, as well as an indicator of the variations in the muscular component during and after chemotherapy [48]. It is also possible that all women treated with NACT for breast cancer undergo breast MRI before and after treatment, while not all women undergo CT. Because of the importance of sarcopenia and sarcopenic obesity, known as risk factors and as factors conditioning the prognosis of oncological diseases, numerous nutritional corrective tools are routinely used by oncologists with the support of nutritionists in order to correct these conditions.

The aim of this work is to confirm the recently introduced role of the pectoralis muscle, quantified on breast MRI as a body composition parameter, as a possible tool for detecting sarcopenia and a guide to the appropriate insertion of nutritional interventions aimed at reducing chemotherapy toxicity and improving outcomes.

The population in this study is mostly made up of women between 50 and 69 years (58.62%), with a body mass index corresponding to normal weight parameters in 46.55% of cases and to slightly overweight parameters in 33.62%. It is, therefore, a rather homogeneous population, which does not present peculiar characteristics in terms of age or obesity, nor particular risk factors predisposing to breast cancer.

On MRI images and, in particular, on T1-weighted ones, the pectoralis muscle is clearly identifiable and easily segmented with a dedicated tool to calculate its overall area. It was, therefore, possible to identify the basal situation of the pectoralis muscle and the muscular area after chemotherapy and consequently calculate the delta, i.e., the difference between the pre- and post-area, indicative of muscle loss during the treatment. In line with previous works by Rossi F. et al., a clear decrease in the pectoralis muscle area measured in MRI was found before and after NACT, with mean bilateral Δ_pect_ values of 2.61 cm^2^ [49]. This difference appeared particularly evident in the subcategory of patients with pre-surgical locoregional staging equal to T2. The loss of muscle mass related to chemotherapy probably has a multifactorial genesis, as already reported by other authors as being both linked to the pharmacokinetics of the therapies and to the establishment of an inflammatory state [48,50]. Furthermore, asthenia, a symptom frequently encountered during chemotherapy, is directly related to the loss of muscle mass; this confirms the importance of rapidly detecting the depletion of the muscle compartment in order to counteract the loss of muscle mass and improve the symptoms of patients [48,50].

A good portion of our patients (85/116) underwent also systemic CT staging before NACT. It was possible to perform a body composition analysis of these patients by calculating both the parameters related to the muscular component and those related to the adipose component. In patients who also had a CT scan pre- and post-neoadjuvant treatment, it was also possible to calculate the Δ skeletal muscle index (SMI), obtaining a mean of 2.16 cm^2^, representative of the variation in the psoas muscle related to the treatments. We, therefore, observed a mean change in the pectoralis muscle similar to the mean change in the psoas muscle: 2.61 cm vs. 2.16 cm^2^.

Therefore, although sarcopenia can cause variations in different entities depending on the body areas in which it is evaluated, these data confirm how it can affect all muscle groups in the same way, and consequently how the changes in the pectoralis muscle are representative of those of the psoas muscle; therefore, we can affirm that the evaluation of the pectoralis muscles is a valid alternative for measuring sarcopenia in patients with breast cancer who only have MR images available [30,48]. Furthermore, the pectoralis areas pre- and post-NACT showed a strong, statistically significant association with all body composition parameters, both related to the muscular compartment and the adipose compartment both pre- and post-chemotherapy. These results reinforce the highly significant correlation between the pectoralis muscle and body composition parameters calculated at CT and indicate how body composition is a balance between different environments closely related to each other. Our work has the advantage of having considered body composition in its complexity, including both the muscular and adipose components.

In line with previous studies [1,2], we used a CT SMI cut-off for sarcopenia of 39 cm^2^/m^2^, according to which we classified the patients who performed a pre-chemotherapy CT scan as sarcopenic (85.88%) or not sarcopenic (14.12%). We identified that, in the majority of the patients, a condition of sarcopenia was already evident at the time of diagnosis of breast cancer. In 41.93% of these patients, we found sarcopenic obesity with a BMI > 25. The pectoralis muscle parameters, pre- and post-NACT, pectoralis delta, and standardized pectoralis index were associated with the evidence of sarcopenia identified in the SMI in a statistically significant way. They are also significantly associated with the condition of sarcopenic obesity. In these patients, the values of the pectoralis muscle area were decidedly lower than in non-sarcopenic patients, with an average bilateral area of 14.88 cm^2^, approximately 21% lower than the average pectoral area of the non-sarcopenic patients (18.94 cm^2^). These data are extremely important, as they not only further strengthen the validity of using the pectoralis muscle in measuring sarcopenia, but also offer the possibility of identifying a threshold to classify patients as sarcopenic using the pectoralis muscle without CT analysis of the muscular parameters of body composition. The optimal cut-off for the pectoralis major area of 20.55 achieves a specificity of 92% to classify into sarcopenia and not sarcopenia. Currently, in fact, the studies available in the literature only focus on variations in the area of the pectoralis muscles, without proposing cut-off values to formulate a diagnosis of sarcopenia in the same way in which this diagnosis is carried out by measuring the SMI in CT [30,48].

In line with the current literature, this study shows an effective decrease in pectoralis muscle area, measured by MRI, after NACT [49]; furthermore, there is a significant correlation between the pectoralis muscle area obtained by MRI and CT parameters of muscle mass, such as psoas muscle area and skeletal muscle tissue area at L3 level. Since sarcopenia is now notoriously considered a negative prognostic factor on multiple levels, and since it is evident that the various composition parameters measured by CT and MRI present significant correlations among them, it is clear that, in the future, MRI may constitute a valid screening tool for sarcopenia in patients with breast cancer.

The high incidence of sarcopenia and sarcopenic obesity for the diagnosis of breast cancer highlights the importance of having reliable tools to be able to already recognize these conditions at diagnosis in order to be able to activate adequate tools for the recovery of these conditions through nutritional and physical activity strategies, to be guaranteed throughout the treatment process in order to also reduce the quota of sarcopenic conditions that occur during treatment.

Some molecular subtypes, such as triple-negative, are significantly associated with lower muscle area values at diagnosis, thus presenting a greater propensity for sarcopenia, a trend also observed in HER2-positive types, compared to luminal-like ones. At the same time, luminal-like subtypes show greater muscle loss during chemotherapy treatment compared to HER2-positive ones. These correlations denote a possible different association between sarcopenia and molecular breast cancer subtype at diagnosis and a differentiated impact on the muscle mass during the treatment. In recent years, nutritional support tools have become an integral part of oncological therapies in order to both prevent the onset of conditions such as sarcopenia or malnutrition, and to reduce the incidence of treatment toxicity. Consequentially, the results that we obtained are potentially very important evidence sources, with a high clinical impact on chemotherapy treatments in cancer patients as they would entail the need to manage nutritional treatments differently in relation to the intensity and time of start depending on the molecular subtype.

Another very strong result is the evidence in patients undergoing a dose-dense chemotherapy regimen of a significant increase in visceral adipose tissue during treatment compared to patients who did not receive a dose-dense regimen. This correlation is not clear in its meaning, as the results in the literature are conflicting. In general, the increase in visceral adipose tissue is associated with oncological treatments that involve the administration of high-dose corticosteroids [51], while the increase in VAT and SAT is associated with worse outcomes in patients with breast cancer [24,52]. Indeed, at our institution, it is common to use twice-weekly paclitaxel 175 mg/m^2^ in dose-dense regimens, which requires more steroid pre-medication than weekly paclitaxel 80 mg/m^2^. This probably explains only part of this association. In general, the accumulation of visceral adipose tissue is a negative predictive factor in terms of survival in oncology [24].

These aspects detected in our work also introduce the possibility of further personalizing nutritional interventions on the basis of the histological breast cancer subtype, and also the chemotherapy regimen, if our specific results can be confirmed in larger case series.

In our opinion, it is definitely advisable to send a sarcopenic patient for dietary counselling. At our institution, we now have the opportunity to request nutritional counselling for patients who experience weight loss during neoadjuvant chemotherapy, in order to draw up individual nutrition plans or possible dietary supplements. This approach is useful in slowing the progression of sarcopenia, reducing the extent of preoperative treatment-related toxicity, maintaining a good quality of life, and at the same time allowing the appropriate dose intensity to be maintained to optimize therapeutic outcomes. However, this approach has its limitations: it only takes weight loss into account as a marker for a state of malnutrition; and the intervention is carried out when the pre-operative treatment is already at an advanced stage, not as a precautionary measure. Based on what we have shown in this paper, identifying sarcopenic patients at the time of diagnosis would allow anticipating the time of nutritional counselling by implementing dietary measures at the start of neoadjuvant treatment. It could also be further tailored based on breast cancer subtype and prescribed chemotherapy regimen to minimize the negative impact of preoperative treatment on the nutritional status of individual patients.

Our work has some limitations, first of all it is a retrospective study, even if on a homogeneous population of women. The patients selected for the study underwent different chemotherapy treatments in relation to different molecular subtypes of breast cancer. This is a limitation, especially in detecting the impact of chemotherapy on body composition. The third limitation is that not all patients selected for the study had undergone staging CT, and the lack of CT scans at the end of chemotherapy treatment reduces the body composition parameters available after treatment. Finally, our results may be susceptible to bias introduced by the lack of adjustment for potential confounding factors.

## 5. Conclusions

The results of our study have highlighted a strong association between body composition parameters and the pectoralis muscle area assessed on breast MRI, both at diagnosis and in relation to changes induced by chemotherapy, indicating that the pectoralis muscle reflects the body muscular compartment. This introduces the possibility of assessing body composition also on breast MRI, a tool widely used in patients with breast cancer undergoing chemotherapy. The novelty compared to studies in the literature is to have estimated on the basis of these correlations a cut-off value that reflects the SMI threshold for the definition of sarcopenia usually used. It is, therefore, possible to recognize sarcopenic patients by analyzing the pectoral muscle area, and therefore obtain important information in the management and nutritional support of the patient with breast cancer during chemotherapy.

Our results also introduced the possibility that there are several correlations between molecular subtypes and sarcopenia, the knowledge of which would allow a better control of the nutritional status of patients in order to prevent malnutrition and make treatment more effective. However, further studies are needed to confirm these results.

## Figures and Tables

**Table 1:** Population characteristics.

Patients’ Characteristics (=116 Patients)		
	**Years**	**N of Patients (%)**
Age at diagnosis	<49	35 (30.17%)
	50–69	68 (58.62%)
	>70	13 (11.21%)
		**N of Patients (%)**
BMI class	Underweight (<18.5)	6 (5.17%)
	Normal weight (18.5–24.9)	54 (46.55%)
	Overweight (25.0–29.9)	39 (33.62%)
	Obesity first class (30.0–34.9)	14 (12.07%)
	Obesity second class (35.0–39.9)	2 (1.72%)
	Obesity third class (>40.0)	1 (0.86%)

**Table 2:** Histological features.

Histological Features of the Diagnostic Samples				
		N of Patients–%		
Type	Invasive ductal carcinoma (IDC)	Grade 2	40	34.48%
		Grade 3	70	60.34%
	Invasive lobular carcinoma (ILC)	Grade 2	4	3.45%
		Grade 3	2	1.72%
Immunohistochemistry	Luminal-like		29	25.00%
	Triple negative		30	25.86%
	HER2+		57	49.14%
Estrogen receptors	Positive		67	57.76%
	Negative		49	42.24%
Progesterone receptors	Positive		56	48.28%
	Negative		60	51.72%
Ki-67/MIB-1	≥20%		98	84.48%
	<20%		18	15.52%
Lymph node status	Positive		51	43.97%
	Negative		65	56.03%

**Table 3:** Baseline local staging and baseline nodal staging.

**Baseline Local Staging**	**N of Patients**	**%**
T1	35	30.17
T2	64	55.17
T3	14	12.07
T4	2	1.72
TX	1	0.86
**Baseline Nodal Staging**	**N of Patients**	**%**
N0	64	55.17
N1	41	35.4
N2	10	8.62
NX	1	0.86

**Table 4:** Neoadjuvant protocols.

Neoadjuvant Protocols	N of Patients	%
Anthracyclines	95	81.9
No Anthracyclines	21	18
Carboplatin	25	21.5
No Carboplatin	91	78.4
Paclitaxel	98	84.4
Docetaxel	18	15.5
Trastuzumab	27	23.2
Trastuzumab + Pertuzumab	25	21.5
No anti-HER2	55	47.4
Immunocheckpointinhibitors	3	2.5
Anti-HER2+ Immunocheckpointinhibitors	6	5

**Table 5:** MRI pectoralis major muscles area, right, left, and bilateral, before and after NACT.

	Pre-NACT				Post-NACT				Δ_pett_			
Parameter	Mean	SD	Min	Max	Mean	SD	Min	Max	Mean	SD	Min	Max
Right PMA	7.66	2.16	4.03	13.92	6.61	1.79	2.51	11.41	1.05	1.04	−0.24	5.47
Left PMA	7.62	2.29	3.3	17.44	6.60	1.88	3.29	1.8	1.03	1.00	−1.06	5.74
Bilateral PMA	15.28	4.19	7.34	30.91	13.21	3.50	5.8	22.87	2.61	6.12	0.03	8.63
PMI (cm^2^/m^2^)	5.79	1.66	2.87	13.38	5.01	1.37	2.27	9.90	0.79	0.68	−0.07	3.48

**Table 6:** Body composition parameters.

	Pre-NACT				Post-NACT				Δ			
Body Composition Parameters	Mean	SD	Min	Max	Mean	SD	Min	Max	Mean	SD	Min	Max
Right psoas area	6.87	1.52	3.77	11.07	6.42	1.48	3.50	9.77	0.77	0.75	−0.05	2.91
Left psoas area	7.07	1.71	3.88	11.33	6.44	1.29	4.02	8.78	0.57	0.47	−0.04	2.06
Bilateral psoas area	1.94	3.13	7.80	22.01	12.86	2.67	7.75	18.55	1.34	1.08	0.05	4.22
Psoas muscle index	5.23	1.14	3.07	8.08	4.75	0.90	3.13	6.81	0.49	0.39	0.02	1.59
Skeletal muscle area	87.94	16.64	44.54	128.10	81.48	14.97	51.21	104.68	5.80	3.60	−1.74	12.79
Skeletal muscle index	32.97	5.91	16.97	47.80	30.09	5.15	20.51	40.31	2.16	1.33	−0.60	4.58
Visceral fat area	79.14	67.04	5.34	256.71	86.78	64.26	11.17	218.98	−2.37	20.58	−43.17	62.16
Visceral fat index	30.24	25.89	1.78	100.67	32.39	23.85	3.82	82.42	−0.80	7.71	−15.48	22.83
Subcutaneous fat area	205.80	112	27.88	485.95	203.05	115.11	35.97	485.76	3.19	47.01	−98.81	145.45
Subcutaneous fat index	78.39	44.16	9.65	189.43	75.90	44.03	12.45	180.01	1.49	17.22	−36.29	55.42
Total fat area	284.94	159.84	35.84	604.48	289.82	161.06	54.85	695.47	0.81	56.64	−106.76	157.71
Total fat index	108.63	62.87	14.00	241.04	108.29	61.22	18.98	255.45	0.68	20.95	−39.21	60.09

**Table 7:** Associations between pectoralis muscle and body composition pre-chemotherapy.

Body Composition Parameters	Pectoralis Muscle Parameters	Mean Difference	95% CI		*p*-Value	R^2^
Right Psoas Area (cm^2^)	Pre-MR bil. pectoralis muscle area (cm^2^)	0.16	0.10	0.23	0.000	0.21
Right Psoas Area (cm^2^)	Pre-MR pectoralis muscle index (cm^2^/m^2^)	0.33	0.15	0.51	0.000	0.14
Left Psoas Area (cm^2^)	Pre-MR bil. pectoralis muscle area (cm^2^)	0.16	0.09	0.24	0.000	0.17
Left Psoas Area (cm^2^)	Pre-MR pectoralis muscle index (cm^2^/m^2^)	0.32	0.12	0.53	0.003	0.10
Bil. Psoas Area (cm^2^)	Pre-MR bil. pectoralis muscle area (cm^2^)	0.33	0.19	0.47	0.000	0.20
Bil. Psoas Area (cm^2^)	Pre-MR pectoralis muscle index (cm^2^/m^2^)	0.66	0.29	1.02	0.001	0.13
Psoas Index (cm^2^/m^2^)	Pre-MR bil. pectoralis muscle area (cm^2^)	0.12	0.07	0.17	0.000	0.21
Psoas Index (cm^2^/m^2^)	Pre-MR pectoralis muscle index (cm^2^/m^2^)	0.30	0.17	0.43	0.000	0.20
Skeletal Muscle Area L3 (cm^2^)	Pre-MR bil. pectoralis muscle area (cm^2^)	1.67	0.91	2.43	0.000	0.18
Skeletal Muscle Area L3 (cm^2^)	Pre-MR pectoralis muscle index (cm^2^/m^2^)	3.12	1.13	5.10	0.003	0.10
Skeletal Muscle Index (cm^2^/m^2^)	Pre-MR bil. pectoralis muscle area (cm^2^)	0.63	0.36	0.89	0.000	0.20
Skeletal Muscle Index (cm^2^/m^2^)	Pre-MR pectoralis muscle index (cm^2^/m^2^)	1.53	0.86	2.20	0.000	0.19
Visceral Fat Area (cm^2^)	Pre-MR bil. pectoralis muscle area (cm^2^)	6.07	2.95	9.20	0.000	0.15
Visceral Fat Area (cm^2^)	Pre-MR pectoralis muscle index (cm^2^/m^2^)	16.73	9.08	24.37	0.000	0.18
Visceral Fat Index (cm^2^/m^2^)	Pre-MR bil. pectoralis muscle area (cm^2^)	2.31	1.10	3.52	0.000	0.14
Visceral Fat Index (cm^2^/m^2^)	Pre-MR pectoralis muscle index (cm^2^/m^2^)	6.72	3.79	9.64	0.000	0.20
Subcutaneous Fat Area (cm^2^)	Pre-MR bil. pectoralis muscle area (cm^2^)	9.23	3.94	14.53	0.001	0.12
Subcutaneous Fat Area (cm^2^)	Pre-MR pectoralis muscle index (cm^2^/m^2^)	26.37	13.45	39.29	0.000	0.16
Subcutaneous Fat Index (cm^2^/m^2^)	Pre-MR bil. pectoralis muscle area (cm^2^)	3.46	1.36	5.56	0.002	0.11
Subcutaneous Fat Index (cm^2^/m^2^)	Pre-MR pectoralis muscle index (cm^2^/m^2^)	10.59	5.52	15.67	0.000	0.17
Total Fat Area (cm^2^)	Pre-MR bil. pectoralis muscle area (cm^2^)	15.31	7.94	22.67	0.000	0.17
Total Fat Index (cm^2^/m^2^)	Pre-MR bil. pectoralis muscle area (cm^2^)	5.77	2.85	8.69	0.000	0.15
Total Fat Index (cm^2^/m^2^)	Pre-MR pectoralis muscle index (cm^2^/m^2^)	17.31	10.32	24.30	0.000	0.22

**Table 8:** Associations between pectoralis muscle and body composition post-chemotherapy.

Body Composition Parameters	Pectoralis Muscle Parameters	Mean Difference	95% CI		*p*-Value	R^2^
Right Psoas Area (cm^2^)	Post-MR bil. pectoralis muscle area (cm^2^)	0.16	0.02	0.30	0.032	0.15
Left Psoas Area (cm^2^)	Post-MR bil. pectoralis muscle area (cm^2^)	0.13	0.01	0.25	0.048	0.13
Bil. Psoas Area (cm^2^)	Post-MR right pectoralis muscle area (cm^2^)	0.53	0.05	1.02	0.038	0.14
Bil. Psoas Area (cm^2^)	Post-MR left pectoralis muscle area (cm^2^)	0.51	0.04	0.99	0.042	0.13
Bil. Psoas Area (cm^2^)	Post-MR bil. pectoralis muscle area (cm^2^)	0.29	0.04	0.53	0.032	0.15
Psoas Index (cm^2^/m^2^)	Post-MR left pectoralis muscle area (cm^2^)	0.18	0.01	0.34	0.042	0.14
Psoas Index (cm^2^/m^2^)	Post-MR bil. pectoralis muscle area (cm^2^)	0.09	0.01	0.18	0.042	0.13
Skeletal Muscle Area L3 (cm^2^)	Post-MR bil. pectoralis muscle area (cm^2^)	2.32	1.07	3.57	0.001	0.31
Skeletal Muscle Area L3 (cm^2^)	Post-MR pectoralis muscle index (cm^2^/m^2^)	4.87	1.21	8.52	0.014	0.19
Skeletal Muscle Index (cm^2^/m^2^)	Post-MR bil. pectoralis muscle area (cm^2^)	0.78	0.35	1.21	0.001	0.30
Skeletal Muscle Index (cm^2^/m^2^)	Post-MR pectoralis muscle index (cm^2^/m^2^)	2.08	0.91	3.26	0.002	0.29
Visceral Fat Area (cm^2^)	Post-MR bil. pectoralis muscle area (cm^2^)	7.92	2.12	13.72	0.012	0.20
Visceral Fat Area (cm^2^)	Post-MR pectoralis muscle index (cm^2^/m^2^)	22.33	6.89	37.76	0.008	0.22
Visceral Fat Index (cm^2^/m^2^)	Post-MR left pectoralis muscle area (cm^2^)	5.82	1.77	9.86	0.009	0.22
Visceral Fat Index (cm^2^/m^2^)	Post-MR bil. pectoralis muscle area (cm^2^)	2.83	0.65	5.00	0.016	0.18
Visceral Fat Index (cm^2^/m^2^)	Post-MR pectoralis muscle index (cm^2^/m^2^)	8.51	2.82	14.20	0.007	0.23
Subcutaneous Fat Area (cm^2^)	Post-MR bil. pectoralis muscle area (cm^2^)	13.46	2.94	23.97	0.018	0.18
Subcutaneous Fat Area (cm^2^)	Post-MR pectoralis muscle index (cm^2^/m^2^)	40.35	12.77	67.94	0.008	0.22
Subcutaneous Fat Index (cm^2^/m^2^)	Post-MR left pectoralis muscle area (cm^2^)	9.77	2.13	17.41	0.018	0.18
Subcutaneous Fat Index (cm^2^/m^2^)	Post-MR bil. pectoralis muscle area (cm^2^)	4.86	0.79	8.93	0.026	0.16
Subcutaneous Fat Index (cm^2^/m^2^)	Post-MR pectoralis muscle index (cm^2^/m^2^)	15.64	5.13	26.15	0.007	0.23
Total Fat Area (cm^2^)	Post-MR bil. pectoralis muscle area (cm^2^)	21.37	7.12	35.62	0.006	0.23
Total Fat Area (cm^2^)	Post-MR pectoralis muscle index (cm^2^/m^2^)	62.68	25.38	99.99	0.003	0.27
Total Fat Index (cm^2^/m^2^)	Post-MR bil. pectoralis muscle area (cm^2^)	7.68	2.18	13.18	0.010	0.21
Total Fat Index (cm^2^/m^2^)	Post-MR pectoralis muscle index (cm^2^/m^2^)	24.15	10.04	38.26	0.002	0.28

**Table 9:** Associations between the pectoralis muscle parameters and sarcopenia.

Outcome	Pectoralis Muscle Parameters	OR	95% CI		*p*-Value
Evidence of Sarcopenia	Pre-MR right pectoralis muscle area (cm^2^)	0.67	0.50	0.90	0.008
Evidence of Sarcopenia	Pre-MR left pectoralis muscle area (cm^2^)	0.73	0.57	0.92	0.009
Evidence of Sarcopenia	Pre-MR bil. pectoralis muscle area (cm^2^)	0.81	0.70	0.94	0.006
Evidence of Sarcopenia	Pre-MR pectoralis muscle index (cm^2^/m^2^)	0.61	0.43	0.88	0.008
Evidence of Sarcopenia	Post-MR right pectoralis muscle area (cm^2^)	0.61	0.41	0.89	0.012
Evidence of Sarcopenia	Post-MR left pectoralis muscle area (cm^2^)	0.54	0.38	0.78	0.001
Evidence of Sarcopenia	Post-MR bil. pectoralis muscle area (cm^2^)	0.72	0.59	0.89	0.002
Evidence of Sarcopenia	Post-MR pectoralis muscle index (cm^2^/m^2^)	0.44	0.26	0.74	0.002
Obesity + Sarcopenia	Pre-MR left pectoralis muscle area (cm^2^)	1.74	1.08	2.81	0.024
Obesity + Sarcopenia	Pre-MR bil. pectoralis muscle area (cm^2^)	1.27	1.01	1.59	0.040
Obesity + Sarcopenia	Pre-MR pectoralis muscle index (cm^2^/m^2^)	2.08	1.09	3.98	0.027
Obesity + Sarcopenia	Post-MR bil. pectoralis muscle area (cm^2^)	1.28	1.00	1.63	0.047
Obesity + Sarcopenia	Post-MR pectoralis muscle index (cm^2^/m^2^)	2.05	1.05	3.98	0.034

**Table 10:** Confusion matrix before NACT.

		Sarcopenia	
		No	Yes
Bilateral pectoralis muscles area	≥20.55	6	6
	<20.55	6	67

## Data Availability

Data are available on request from the authors. The data are not publicly available for privacy.

## Abbreviations

The following abbreviations are used in this manuscript:

PMA	Pectoralis muscle area
NACT	Neoadjuvant chemotherapy
MRI	Magnetic resonance imaging
CT	Computed tomography
BMI	Body mass index
SDI	Standard dose intensity
DDI	Delivered dose intensity
RDI	Relative dose intensity
PMI	Pectoralis muscle index
SMA	Skeletal muscle area
SMI	Skeletal muscle index
HU	Hounsfield units
ROI	Regions of interest
VFA	Visceral fat area
VFI	Visceral fat index
SFA	Subcutaneous fat area
SFI	Subcutaneous fat index
MD	Mean difference
OR	Odds ratios
RRR	Relative risk ratios
CI	Confidence intervals
ROC	Receiver operating characteristic curve
AUC	Area under the curve
SD	Standard deviation
